# GP73 N-glycosylation at Asn144 reduces hepatocellular carcinoma cell motility and invasiveness

**DOI:** 10.18632/oncotarget.8120

**Published:** 2016-03-16

**Authors:** Kai Jiang, Wei Li, Qinle Zhang, Guoquan Yan, Kun Guo, Shu Zhang, Yinkun Liu

**Affiliations:** ^1^ Liver Cancer Institute, Zhongshan Hospital, Fudan University, Key Laboratory of Carcinogenesis and Cancer Invasion, Ministry of Education, Shanghai, China; ^2^ Cancer Research Center, Institutes of Biomedical Sciences, Fudan University, Shanghai, China; ^3^ Interdisciplinary Research Center on Biology and Chemistry, Shanghai Institute of Organic Chemistry, Chinese Academy of Sciences, Shanghai, China

**Keywords:** Golgi protein 73, N-glycan, hepatocellular carcinoma

## Abstract

Golgi Protein 73 (GP73) is a potential liver disease glycobiomarker warranting comprehensive analyses of its glycan structure and glycosylation function. In this study, we used mass spectrometry to identify glycosylation sites and the glycan structure, high-throughput lectin microarray to provide rapid and sensitive profiling of glycoconjugates, and site-directed mutagenesis to clarify the impact of glycans on target glycoproteins *in vivo*. We identified three GP73 N-glycosylation sites: Asn109, Asn144 and Asn398. We found five glycoforms on Asn144, including biantennary, triantennary and fucosylated glycans. Removal of N-glycans at Asn144 enhanced the motility and invasiveness of hepatocellular carcinoma cells, possibly due to inhibition of cell adhesion related to the changes of cell membrane glycosylation. This study increases our understanding of the functional relevance of GP73 glycosylation and suggests that Asn144-deleted GP73 can influence the progression and metastasis of hepatocellular carcinoma.

## INTRODUCTION

Hepatocellular carcinoma (HCC) is the fifth most common cancer and the third leading cause of cancer mortality globally [1, 2] with a poor 5-year survival rate due to tumor recurrence and metastasis [3]. The high incidence of metastasis remains the main obstacle to treatment efficacy. Elucidation of the detailed mechanism of HCC cell growth and metastasis is crucial to improve HCC therapeutic intervention.

GP73, a resident Golgi-specific membrane protein, is often up-regulated in hepatocytes [4–6]. Block et al. found GP73 was elevated and hyperfucosylated in HCC patients (and also woodchucks) using targeted glycoproteomics [7]. Combining differential lectin-based glycoprotein capture with mass spectrometry (MS) analysis, Drake et al. discovered that the serum level of fucosylated GP73 varied with HCC disease state [8]. GP73 was also linked to HCC through precise large scale identification of core-fucosylated glycoproteins with low- and high-normalized collision energy [9]. Norton et al. used conventional lectin affinity chromatography and mass spectrometry to identify glycosylation sites and glycan structure of GP73 secreted from cultured hepatoma cells [10]. Comprehensive analysis of the GP73 glycan structure and glycosylation function could provide additional insight into its role in HCC progression and provide a new avenue for therapeutic intervention.

Protein glycosylation plays a pivotal role in various biological processes such as ligand-receptor binding, immune response, apoptosis, development, and disease. Some elements of tumor biology, such as metastasis and cell survival, are linked to the presence of specific cellular glycoforms [11]. Due to the large diversity and heterogeneity of glycans synthesized in the Golgi, many of their functions are extremely difficult to study and remain unclear [12].

Information on glycan composition, structure, and glycosylation sites can be determined using MS-based methods. MS combined with peptide-N-glycosidase (PNGase F)-mediated incorporation of ^18^O is commonly used to uncover glycosylation sites [13]. Qualitative and quantitative analyses of released glycans or glycopeptides can be performed using Matrix-Assisted Laser Desorption/Ionization (MALDI)-MS and liquid chromatography (LC)-MS [14, 15]. Lectin microarray is a high-throughput technique for rapid and sensitive glycoconjugate profiling, that allows direct analysis of glycoconjugates such as glycoproteins without liberation of glycans from the core substrate [16, 17].

Knockdown or knockout of glycosyltransferase [18] and inhibition of enzymes involved in N-linked glycosylation [19] are common methods to explore the functional relevance of glycosylation. Site-directed mutagenesis can reveal the biological significance of specific target glycoproteins. Mutant N-glycosylation site constructs help to clarify the functional roles of glycans *in vivo* [20]. For example, mutation of CD10 at Asn628 to prevent N-glycosylation greatly decreases its surface expression in HEK293 cells and completely abolishes its neutral endopeptidase activity [21]. Elimination of N-glycans increases the lipolytic activity of apoptosis inhibitor of macrophage (AIM) by enhancing AIM incorporation into adipocytes [22].

In this study, a FLAG-GP73 expression plasmid was constructed and transfected into HCC cells. Recombinant GP73 was immunoprecipitated using its FLAG tag and then LC-electrospray ionization (ESI)-MS was used to identify its N-glycosylation sites. N-glycosylation defective site mutants of GP73 were constructed for functional analysis of the biological significance of its N-glycosylation.

## RESULTS

### Analysis of N-glycosylation site in recombinant GP73

**Figure 1:**
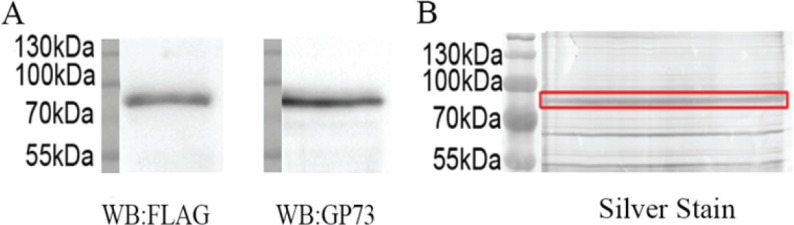
Purification of recombinant GP73 from SMMC7721 (**A**) Western blot analysis of immunoprecipitated GP73. (**B**) The immunoprecipitated GP73 were electrophoresed on SDS-PAGE and silver stained.

The FLAG-GP73 plasmid was constructed and transfected into SMMC7721 cells. Recombinant GP73 was immunoprecipitated using its FLAG tag (Figure 1), electrophoresed on SDS-PAGE, excised from gels, trypsinized, and analyzed by LC-MS/MS, confirming its identity (Supplementary Figure S1).

**Figure 2:**
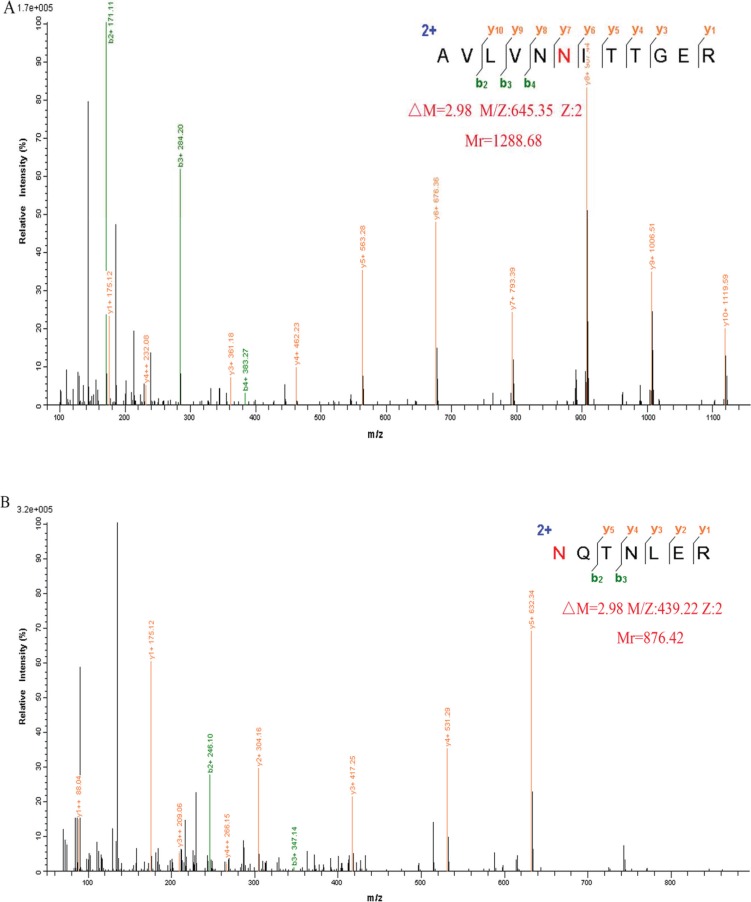
Representative MS/MS spectrum of the peptide AVLVNN^109^ITTGER (A) and N^144^QTNLER (B) of GP73, identified in H_2_^18^O Increased mass of 2.98 Da is indicated.

The trypsinized peptides were processed by PNGase F in H_2_^18^O. PNGase F specifically cleaves N-glycans from the polypeptide backbone and at the same time converts asparagine to aspartic acid. The resulting 2.98 Da mass shift in the presence of H_2_^18^O [23, 24] allows identification of the N-glycosylation site. Two N-glycosylation sites Asn109 and Asn144 were identified by LC-MS/MS. Figure 2 shows representative MS/MS spectra of the peptides AVLVNN^109^ITTGER and N^144^QTNLER of GP73 in which the 2.98 Da mass increase is indicated.

**Figure 3:**
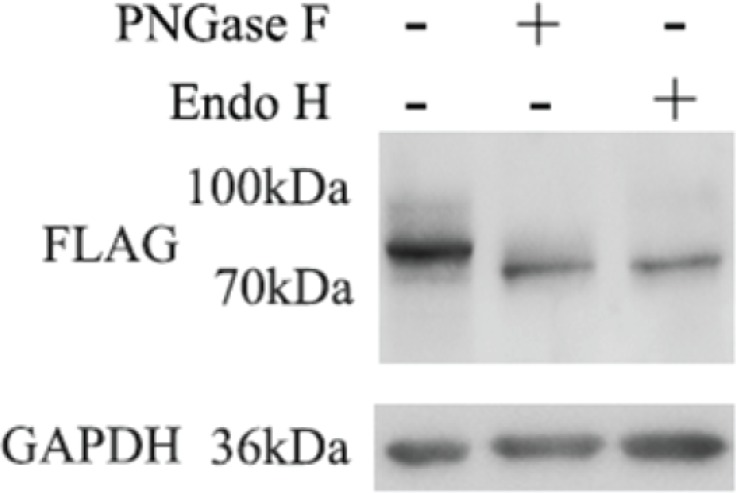
Asn109, 144-deleted GP73 was digested with PNGase F and Endo H, respectively The existence of Asn398 was confirmed by a 7 kDa mass decrease by gel migration.

Manual assertion of the glycosylation rules (Asn-Xxx-Ser/Thr, (Xxx ≠ Pro)) indicates GP73 may have a third N-glycosylation site near its C-terminus. However, only two N-glycosylation sites were detected by LC-MS/MS. This third deglycosylated peptide may not be suitable for LC-MS/MS analysis due to its length or a hydrophobic nature. Site-directed mutagenesis was applied to construct Asn109 and 144-deleted GP73. The existence of Asn398 was confirmed via PNGase F and Endo H digestion and a 7 kDa reduction in mass observed by gel migration (Figure 3).

### Mutated Asn144 enhances HCC cell motility and invasiveness

**Figure 4:**
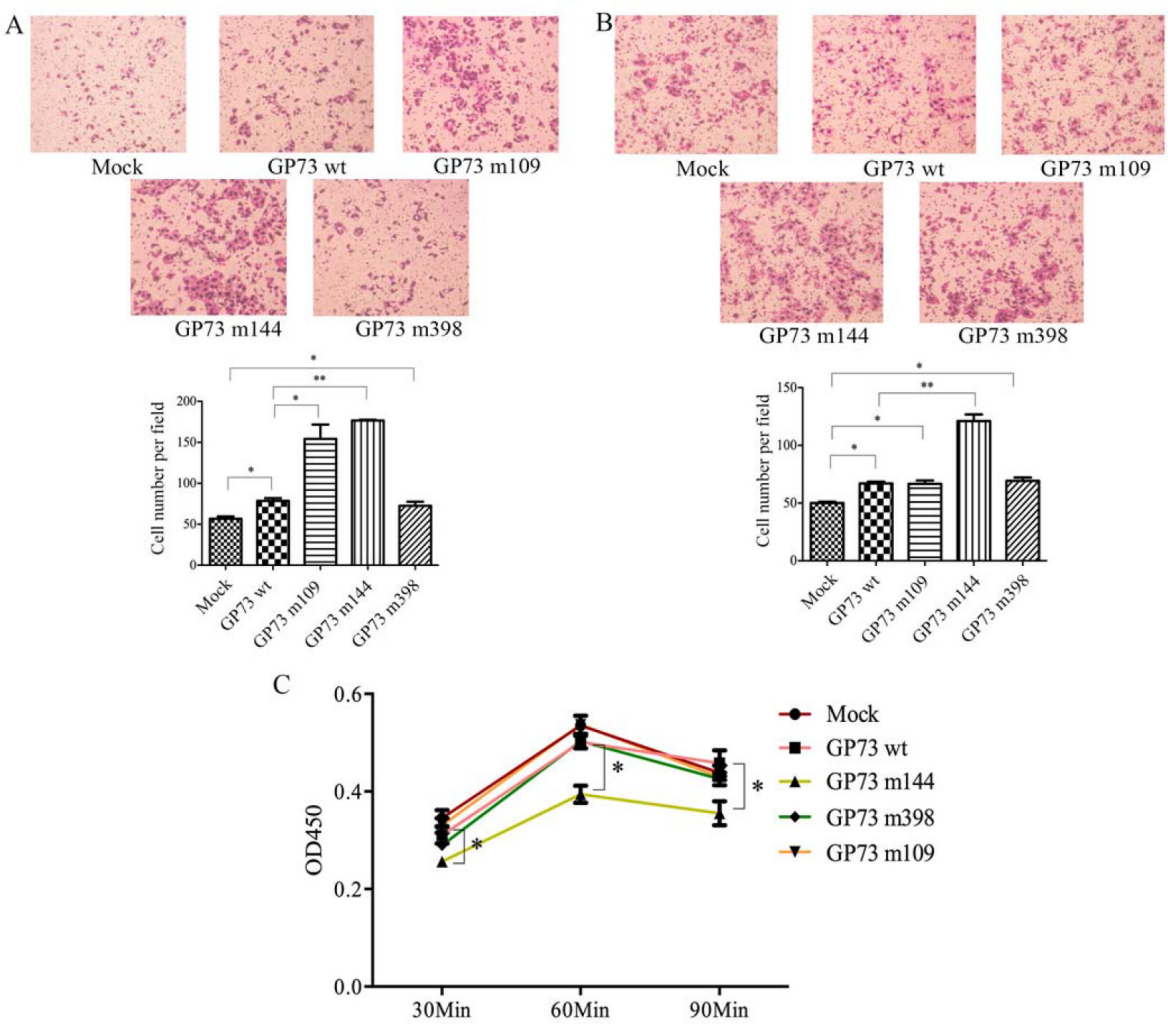
Compared with control group, GP73 m109 and m144 improved cell motility (A) and GP73 m144 enhanced the invasiveness (B) and inhibited adhesion (C)

Site-directed mutagenesis was used to obtain GP73 m109, GP73 m144 and GP73 m398 (Supplementary Figure S2). Wild type GP73 (GP73 wt) and empty vector (Mock) were used as controls. The plasmids were transfected into SMMC7721 cells. Cell migration was tested using a transwell assay chamber; cells that migrated into the lower compartment of the chamber were fixed and then stained with Giemsa. GP73 m109 and GP73 m144 cells had markedly increased motility, showing more migrating cells than GP73 wt and Mock transfected cells (Figure 4A). Invasive activity was also determined using invasion chamber assays with matrigel, which showed that GP73 m144 improved invasive ability (Figure 4B). Cell adhesion assay using laminin indicated that GP73 m144 inhibited adhesion ability compared with GP73 wt and Mock transfected cells (Figure 4C). Taken together, these results indicate that GP73 m109 and GP73 m144 enhanced HCC cell motility; specifically, GP73 m144 both improved invasive ability and inhibited adhesion ability. Properties of HCC cells transfected with GP73 m109, m144, and m398 detected in this study are summarized in Table 1.

**Table 1:** Summary of functional alterations of HCC cells transfected with GP73 m109, m144, and m398*

Cell functions	GP73 m109		GP73 m144		GP73 m398	
Control groups	Mock	GP73 wt	Mock	GP73 wt	Mock	GP73 wt
Cell proliferation	–	–	–	–	–	–
Cell motility	+	+	+ +	+ +	+	+
Cell invasion ability	+	+	+ +	+ +	+	–
Cell adhesion ability	–	+	+	+	–	–

“–” not affected,“+” affected, “++” affected in a large scale.

* Compared with the Mock group, the cell motility and invasion ability of GP73 wt group were “+”.

**Figure 5:**
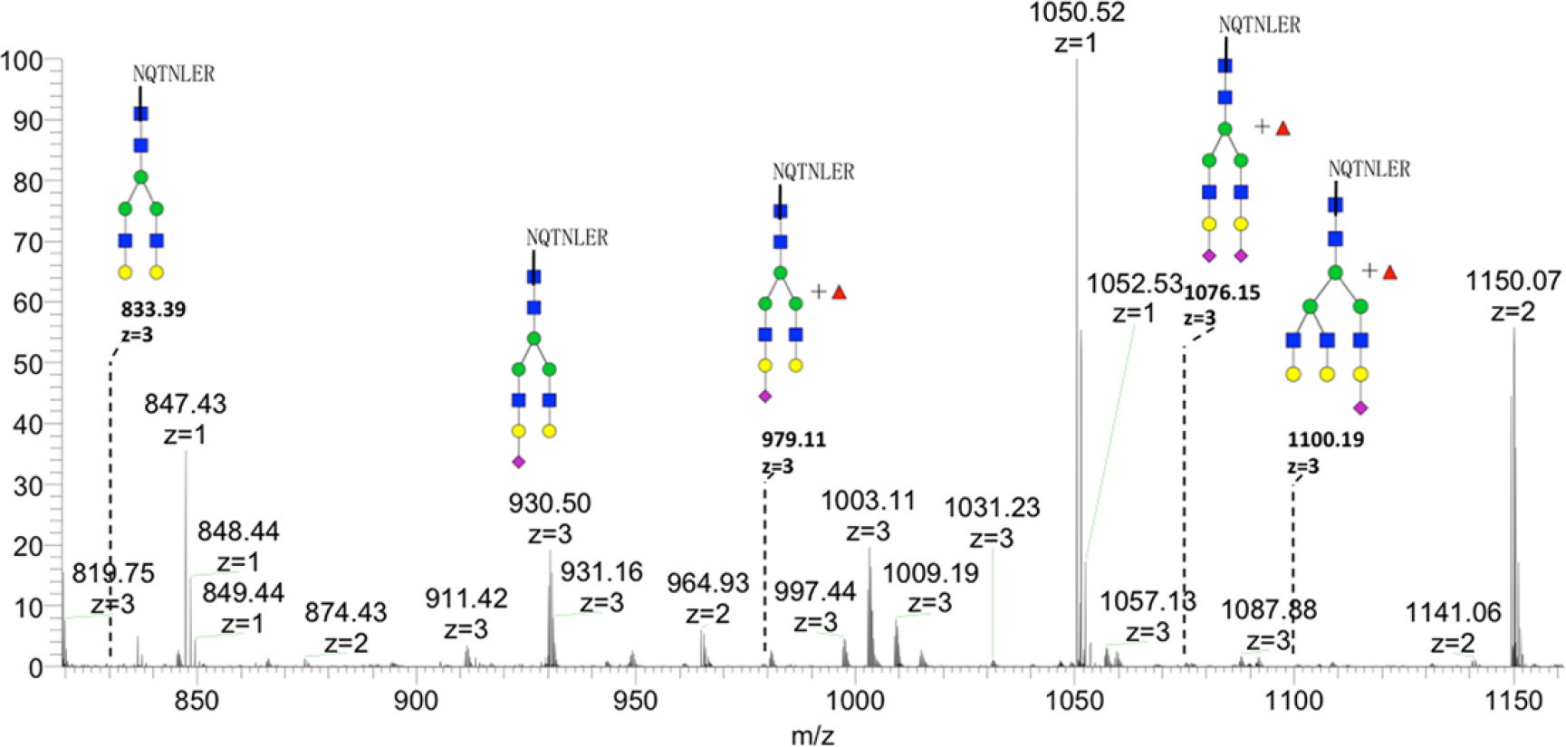
Glycan spectrum of Asn144 on recombinant GP73 by LC-MS Five glycoforms including biantennary, triantennary, and fucosylated glycans were detected.

GP73 Asn144 site-specific glycan structures were analyzed by LC-MS/MS. Tandem MS was used to assign the structure of the glycans and characteristic glycan fragments (m/z 204, 366, 528) were observed in these spectra. There were 5 glycoforms found on Asn144, including biantennary, triantennary, and fucosylated glycans (Figure 5).

### Asn 144-deleted GP73 altered glycoprofiling of membrane protein

Glycans participate in many key biological processes including cell adhesion, molecular trafficking and clearance, receptor activation, signal transduction, and endocytosis [25]. Asn144-deleted GP73 inhibited adhesion ability and increased the invasiveness of HCC cells. To probe the differences in glycan processing of glycoproteins in SMMC7721 cells transfected with GP73 m144, lectin microarray was carried out to uncover alterations of the glycan pattern of cell surface membrane glycoproteins. The lectin microarray preparation protocol was described in our previous publication [26, 27]. Data were collected from three independent measurements, which had similar trends.

**Figure 6:**
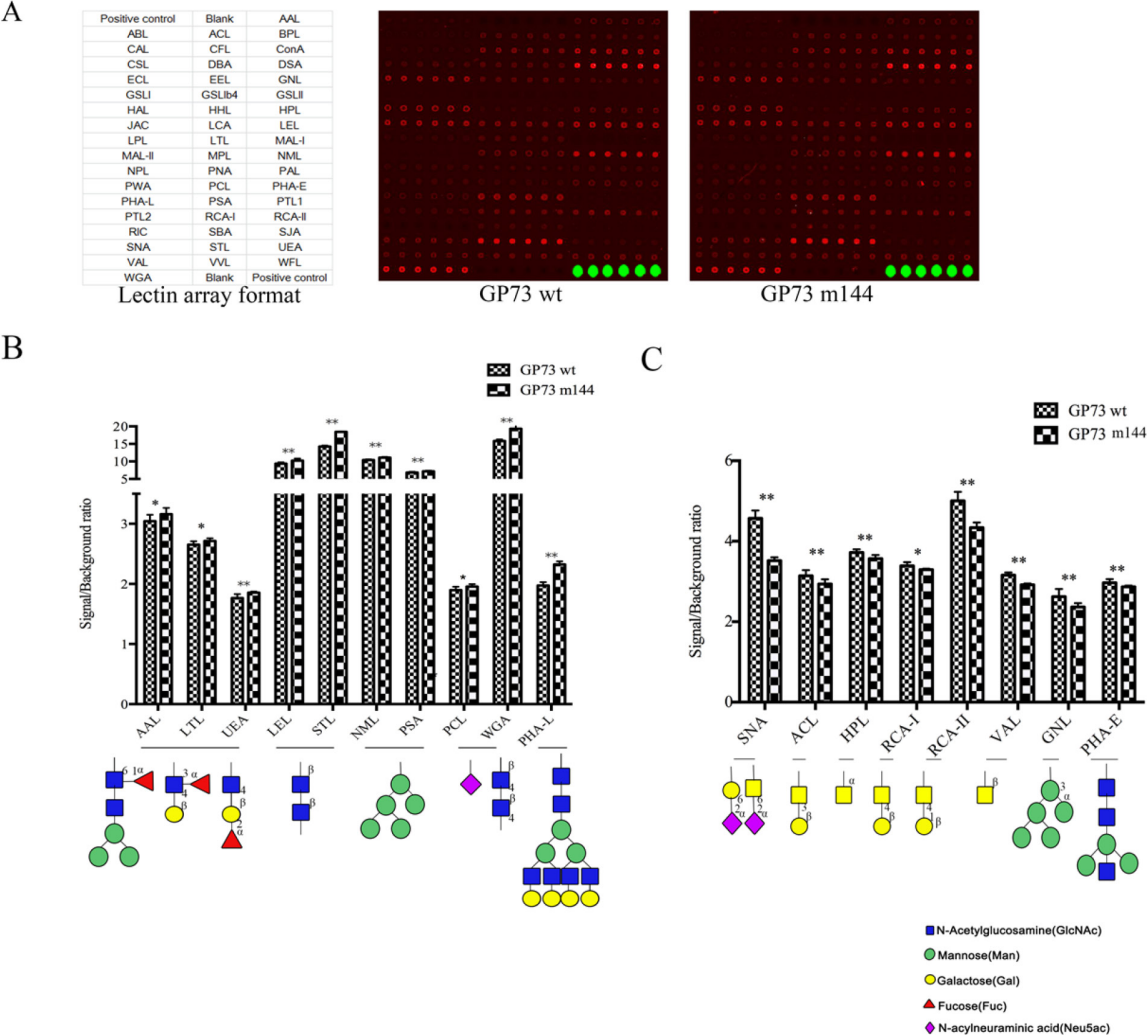
The binding affinity to surface membrane glycoproteins of SMMC7721 cells transfected with GP73 m144 and GP73 wt was analyzed by lectin microarray containing 50 kinds of lectins (**A**) Lectins with different binding specificities and lectin microarray scanned with fluorescence scanner. (**B**) Lectins for profiling the increased cell surface glycoforms in GP73 m144 by bar graph based on lectin microarray data. (**C**) Lectins for profiling the reduced cell surface glycoforms in GP73 m144 by bar graph based on lectin microarray data. Data are the average ± SD of three independent measurements. *represent *p* < 0.05, **represent *p* < 0.01.

Scan images of the cells for all 50 lectins are shown in Figure 6. Normalized signal intensity divided by background intensity was used to evaluate binding affinity of lectins. 18 lectins (Table 2 and Table 3) differed between GP73 m144 and GP73 wt transfected into SMMC7721 cells. Asn144-deleted GP73 reduced α/β linked galactose, N-acetylgalactosamine residues, α1, 3-mannose residues, and bisecting residues of cell surface membrane glycoproteins. Core fucosylation, Lewis x, β linked N-acetylglucosamine, high mannose residues, sialic acid, and β1, 6-branching residues were increased (Figure 6). The absence of Asn144 enhances motility and invasion of HCC cells, possibly due to the inhibition of cell adhesion ability by the changes of cell membrane glycosylation.

**Table 2:** The increased glycan structures recognized by 10 lectins

Lectins	Monosaccharide specificity	Preferred glycan structure (terminal epitope)
AAL (Aleuria aurantia lectin)	Fuc	Fucα6GlcNAc (core Fuc), Fucα3(Galβ4)GlcNAc (Le^x^)
UEA (Ulex europaeus agglutinin)	Fuc	Fucα2Galβ4GlcNAc (H-type 2)
LTL (Lotus tetragonolobus lectin)	Fuc	Fucα3(Galβ4)GlcNAc (Le^x^), Fucα2Galβ4GlcNAc (H-type 2)
PSA (Pisum sativum agglutinin)	Fuc/Man	Fucα6GlcNAc, High-man
NML (Naja mossambica lectin)	Man	High-man including Manα6(Manα3)Man, exopolysacchride
PCL (Phaseolus coccineus lectin)	Sia	Sialic acid
STL (Solanum tuberosum lectin)	GlcNAc	(GlcNAcβ)_n_, (GlcNAcβ4MurNAc)_n_(peptidoglycan backbone)
LEL (Lycopersicon esculentum lectin)	GlcNAc	(GlcNAcβ4)_n_, (Galβ4GlcNAc)_n_(polylactosamine)
WGA (Wheat germ agglutinin)	GlcNAc	(GlcNAcβ4)_n_, NeuAc
PHA-L (Phaseolus vulgaris leucoagglutinin)	Complex	β-1, 6 branching tri/tetra-antennary complex-type N-glycan

**Table 3:** The decreased glycan structures recognized by 8 lectins

Lectins	Monosaccharide specificity	Preferred glycan structure (terminal epitope)
ACL (Amaranthus caudatus lectin)	Gal	Galβ3GalNAc
PHA-E (Phaseolus vulgaris erythroagglutinin)	Gal	N-glycans with outer Gal and bisecting GlcNAc
RCA I (Ricinus communis agglutinin I)	Gal	Galβ4GlcNAc
HPL (Helix pomatia lectin)	GalNAc	GalNAc
RCA II (Ricinus communis agglutinin II)	Gal/GalNAc	Lac/LacNAc, Terminal Galβ1–4 GlcNAc
VAL (Viscum album lectin)	βGalNAc	β-D-galactosyl residues
GNL (Galanthus nivalis lectin)	Man	High-man including Manα3Man
SNA (Sambucus nigra lectin)	Sia	Siaα2–6Gal/GalNAc

## DISCUSSION

Three GP73 N-glycosylation sites Asn109, Asn144, and Asn398 were identified in this study. Glycosylation-deficient site mutants m109, m144 and m398 were transfected into SMMC7721 cells to explore the function of N-glycosylation of GP73. GP73 wt and Mock transfections were used as controls. As shown in Figure 4, both the motility and invasive ability of GP73 wt were increased compared with the Mock cells. Previous reports showing that GP73 promotes HCC cell migration [28] and invasion [29] are consisted with our study. Compared with two control groups, removal of N-glycans at either Asn109 or Asn144 improved cell motility and removal of N-glycans at Asn144 enhanced invasive ability and inhibited adhesion ability. Removal of N-glycans at Asn398 caused no change in cell function. For target glycoproteins, the functions of specific-site glycosylation are often diverse. For example, Zhou et al. revealed that N-glycosylation at Asn633 is essential for E-cadherin expression, folding and trafficking [30]. Zhao et al. reported that N-glycans at Asn554 and Asn566 of E-cadherin, but not at Asn618, are indispensable for E-cadherin-mediated suppression of cell cycle progression. Removal of N-glycans at either Asn554 or Asn566 of E-cadherin was accompanied with the activation of the extracellular signal-regulated protein kinase signaling pathway [31].

Cell motility and invasiveness are closely related with adhesion; in this study, absence of N-glycans at Asn144 enhanced motility and invasiveness of HCC cells by inhibition of adhesion ability. In addition, HCC tissues were collected and sequenced to determine the mutation state of Asn144 of GP73 in this study. This site of GP73 was not mutated in these samples (Supplementary Figure S3), indicating that glycosylation on Asn144, not the site itself, influenced HCC progression and metastasis. Aberrant glycosylation of cell surface glycoproteins is important for adhesion of cancer cells [32]. Thus, high-throughput lectin microarray was chosen to analyze glycosylation of cell membrane proteins in SMMC7721 cells transfected with GP73 m144. The results show that α/β linked galactose, N-acetylgalactosamine residues, α1, 3-mannose residues, and bisecting residues of cell surface membrane glycoproteins were reduced. However, core fucosylation, Lewis x, β linked N-acetylglucosamine, high mannose residues, sialic acid, and β1, 6-branching residues were increased.

β1, 6-branching glycan structures are synthesized by N-acetylglucosaminyltransferase (GnT) -V and bound specifically by Phaseolus vulgaris Leucoagglutinin (PHA-L) lectin. Malignant transformation of human cells is commonly associated with increased β1, 6-branching glycans and their increased presence in primary tumors may be diagnostic of metastatic disease [33–35]. Guo et al. found branched N-glycan expression in the EC2 and EC3 domains of N-cadherin, products of GnT-V, regulated N-cadherin-mediated cell-cell contact formation, outside-in signaling, and cell migration. They concluded that it was a significant contributor to the increased migratory/invasive phenotype of cancer cells [36]. β1, 6-branching glycan may be glycosylated with fucose and sialic acid to induce sialyl Lewis x structure. It has been reported that GnT-V induces sialyl Lewis x expression, leading colon cancer cells to metastasize by enhancing their ability to attach to vascular endothelium in distant organs, such as liver or lung. Inhibition of GnT-V activity may prevent metastasis in colon cancer patients with high sialyl Lewis x expression [37]. Bisecting glycan structures are synthesized by GnT-V, which transfers GlcNAc to the 4-position of core mannose. It is thought that the bisecting structure is not further glycosylated under physiological conditions, in sharp contrast to the GlcNAc at the mannoses on both 1–3 and 1–6 branches [38]. Thus, decreased bisecting and increased Lewis x, β1, 6-branching structures of cell surface membrane glycoproteins correlated strongly with motility and invasiveness of HCC cells.

α2, 6 sialylation was decreased in SMMC7721 cells transfected with GP73 m144 based on SNA-binding, while sialic acid was increased. Sialic acid includes three principle configurations: α2, 3 sialylation, α2, 6 sialylation and α2, 8 sialylation [39]. Thus, α2, 3 sialylation and α2, 8 sialylation might be elevated. Alteration of sialic acid processing in malignant cancer cell leads to a general upregulation of sialylated glycans (hypersialylation) on the cell surface [40]. Kawamoto et al. revealed the localization of alpha 1, 6-fucosyltransferase (Fut8) in the Golgi apparatus was important for the increased GP73 expression. They demonstrated that GP73 regulation through overexpression of a glycosyltransferase may lead to Golgi stress [41]. Most glycosyltransferases are Golgi type II transmembrane proteins with a single transmembrane region. Glycosylation of GP73, particularly, Asn144-deleted GP73 may cooperate with glycosyltransferases to alter cell membrane glycans followed by inhibition of cell adhesion and increased motility and invasiveness. Based on the important role of Asn144 in motility and invasion, it is reasonable to envision a complex regulatory network involving glycosylation and metastasis in HCC. The mechanisms underlying this correlation remain to be investigated in our further study.

In summary, we have identified three N-glycosylation sites on GP73 Asn109, Asn144, and Asn398. Removal of N-glycans at either Asn109 or Asn144 improves HCC cell motility. The absence of Asn144 enhances motility and invasiveness of HCC cells, possibly due to inhibition of cell adhesion by the changes of cell membrane glycosylation. We found 5 glycoforms on Asn144, including biantennary, triantennary, and fucosylated glycans. This study demonstrates that Asn144-deleted GP73 increases HCC progression and metastasis, and enhances our understanding of the functional consequences of GP73 glycosylation.

## MATERIALS AND METHODS

### Cell culture

SMMC7721 was purchased from Chinese Academy of Sciences, Shanghai, China. The cells were cultured in DMEM supplemented with 10% fetal bovine serum at 37°C with 5% CO_2_.

### GP73 purification and western blot

SMMC7721/GP73-FLAG transfected cells were lysed in lysis buffer at 4°C for 30 min; insoluble material was removed by centrifugation at 12,000 g for 30 min. Anti-FLAG antibody-conjugated agarose gel (M2, Sigma, USA) was incubated with the cell lysates overnight under constant agitation at 4°C. After incubation, the anti-FLAG antibody beads were washed three times in lysis buffer to eliminate non-specific binding. Then, beads were incubated with 200 μl 1 × Flag peptide for 30 min with constant agitation at 4°C. Immunoprecipitates were resolved by SDS-PAGE under reducing conditions using 10% gels. After electrophoresis, proteins were transferred to PVDF membrane and probed with the appropriate antibody followed by HRP-conjugated secondary antibody.

### Identification of N-glycosylation sites by LC-MS/MS

GP73 samples were fractionated by 10% SDS-PAGE and protein bands were visualized by silver staining. The 73 kDa bands were excised, destained, reduced, and alkylated. After trypsin (sequencing grade, Promega, USA) digestion, the peptides were applied to PNGase F diluted in H_2_^18^O. The dried peptides were resuspended with 10 μl solvent A (A: water with 0.1% formic acid; B: ACN with 0.1% formic acid), separated by nanoLC, and analyzed by on-line electrospray tandem mass spectrometry. The experiments were performed on a Nano Aquity UPLC system (Waters Corporation, Milford, MA) connected to a quadrupole-Orbitrap mass spectrometer (Q-Exactive) (Thermo Fisher Scientific, Bremen, Germany) equipped with an online nano-electrospray ion source. The Q-Exactive mass spectrometer was operated in the data-dependent mode to switch automatically between MS and MS/MS acquisition. All MS/MS samples were analyzed using Mascot (Matrix Science, London, UK; version 2.3.0.2). Mascot was set up to search the human UniProtKB/Swiss-Prot database (Release 2014_04_14, with 20284 entries). To reduce false positive identification results, a decoy database containing the reverse sequences was appended to the database.

### Characterization of N-glycan structures by LC-MS/MS

The 73 kDa lanes were excised, destained, reduced, and alkylated. After trypsin (sequencing grade, Promega, USA) digestion, sample was loaded onto the trap column (Thermo Scientific Acclaim PepMap C18, 100 μm × 2 cm), with a flow of 10 μl/min for 3 min and subsequently separated on the analytical column (Acclaim PepMap C18, 75 μm × 15 cm) with a linear gradient, from 2% B to 40% B in 105 min. The column was re-equilibrated at initial conditions for 15 min. The column flow rate was maintained at 300 nL/min and column temperature was maintained at 40°C. The electrospray voltage of 1.9 kV versus the inlet of the mass spectrometer was used. The Q-Exactive mass spectrometer was operated in the data-dependent mode to switch automatically between MS and MS/MS acquisition. Survey full-scan MS spectra (m/z 350–1200) were acquired with a mass resolution of 70 K, followed by fifteen sequential high energy collisional dissociation (HCD) MS/MS scans with a resolution of 17.5 K. In all cases, one microscan was recorded using dynamic exclusion of 30 seconds.

### Mutation of N-glycosylation sites by site directed mutagenesis

GP73 N-glycosylation sites were mutated to Gln using sequence overlap extension PCR. The pCR3.1 plasmid/GP73-FLAG was used as the template. The primers used to mutate GP73 N-glycosylation sites were as follows (mutated codon is bolded):

GP73 m109 (Asn109 mutated to Gln109):

Forward: 5′-AAAGGCGGTTTTGGTGAAT**CAA**ATCACCACAGGTGAGAGG-3′

Reverse: 5′-CCTCTCACCTGTGGTGAT**TTG**ATTCACCAAAACCGCCTTT-3′

GP73 m144 (Asn144 mutated to Gln144):

Forward: 5′-GTCCTCCAGTTTCAGAAG**CAA**CAGACCAACCTGGAGAGG-3′

Reverse: 5′-CCTCTCCAGGTTGGTCTG**TTG**CTTCTGAAACTGGAGGAC-3′

GP73 m398 (Asn398 mutated to Gln398):

Forward:

5′-TTGATCAGCGTGAAAAGCGG**CAA**CATACACTCATGAGTAAAGG-3′

Reverse: 5′-CCTTTACTCATGAGTGTATG**TT G**CCGCTTTTCACGCTGATCAA-3′

All mutations were verified by DNA sequencing to ensure the presence of the correct mutation and the absence of any other randomly introduced mutations.

### Plasmid production and HCC cells transfection

The pCR3.1 plasmid was conjugated with GP73 cDNA with FLAG tag at the carboxyl terminus. After digestion with EcoRI and BamHI, the construct was fully sequenced. The plasmids were transfected into SMMC7721 cells using Lipofectamine 2000 (Invitrogen, USA). SMMC7721 cells transfected with plasmids were selected with 1 μg/ml puromycin for at least 7 days before use.

### Transwell migration and invasion assay

Cell migration and invasion assay was performed using 24-well transwell plates (8.0 μm pore size; Millipore, USA) precoated without or with matrigel (BD Biosciences, USA). SMMC7721 cells (5 × 10^4^) were suspended in 1.5 ml serum-free DMEM media and transferred into the inside chamber of a 24-well cell culture insert with 8.0 μm pore size. 600 μl media with 20% FBS was added into the outside well. After 24 h incubation, cells remaining on the upper side of the filters were cleaned with cotton-tipped swabs. Cells on the lower surface of the membrane were fixed by methanol and subjected to Giemsa staining. The cells on the underside of the filters were counted at five randomly selected fields (at 200 × magnification), and the average cell number per view was calculated. All experiments were performed in triplicate.

### Cell adhesion assay

96-well, flat-bottom culture plates were coated with 50 μl laminin (10 μg/ml) (Sigma, USA) in phosphate-buffered saline overnight at 4°C. Plates were then blocked with 0.2% BSA for 2 h at room temperature followed by washing three times with DMEM. The cells were harvested with trypsin/EDTA, washed twice and resuspended in DMEM medium. Cells were added to each well in triplicate and incubated for 30 min, 60 min, or 90 min at 37°C. Plates were then washed three times with DMEM medium to remove unbound cells. Cells remaining attached to the plates were quantified by CCK8 assay at a wavelength of 450 nm.

### Lectin microarray analysis

Membrane proteins were extracted with ProteoExtract Native Membrane Protein Extraction Kit (Merk Millipore, USA) and then biotinylated. An equal amount of biotinylated proteins was applied to the lectin array containing 6 repeated spots of 50 lectins. After 3 h incubation at room temperature, the glass slide was washed 3 times with PBS containing 1% Triton X-100 (PBSTx). 60 μL Cy5-labeled streptavidin (Invitrogen, USA) solution in PBSTx was added to the array and incubated at room temperature for 30 min. The glass slide was rinsed with PBSTx and scanned by an evanescent-field fluorescence scanner (Capitalbio, China). The data was obtained by the Array Pro Analyzer, version 4.5 (Media Cybernetics). The signal intensity of a spot was considered valid when the ratio of spot intensity/background intensity > 1.5. Then, the intensity of each spot was calculated by subtracting the background intensity from the signal intensity. The intensity value for each lectin was the average intensity of 6 repeated spots. The lectin array was repeated in triplicate.

### Statistical analysis

Statistical analysis was performed with SPSS 15.0 for Windows (SPSS, Chicago, IL). Data were presented as the mean ± SD unless otherwise indicated. The Student's test (two tailed) was used to compare two groups of parametric variants, and Spearman's rho test or chi-square test was used to analyze non-parametric variants. *P* < 0.05 was considered statistically significant.

## SUPPLEMENTARY MATERIALS FIGURES


