# Supplementary material for: GP73 N-glycosylation at Asn144 reduces hepatocellular carcinoma cell motility and invasiveness
Source: Oncotarget. 2016 Mar 16;7(17):23530–41. doi: 10.18632/oncotarget.8120 (PMC5029645; doi:10.18632/oncotarget.8120)
Supplement: Supplementary file 1 [file oncotarget-07-23530-s001.pdf]

GP73 N-glycosylation at Asn144 reduces hepatocellular carcinoma cell motility and invasiveness

Supplementary Materials

Protein View

Match to: **GOLM1_HUMAN** Score: **5380**
Golgi membrane protein 1 OS=Homo sapiens GN=GOLM1 PE=1 SV=1
Found in search of D:\redo\0122\YJ-73-1.mgf

Nominal mass (M_r): **45306**; Calculated pI value: **4.91**
NCBI BLAST search of GOLM1_HUMAN against nr
Unformatted sequence string for pasting into other applications

Taxonomy: Homo sapiens

Variable modifications: Acetyl (Protein N-term),Oxidation (M)
Cleavage by Trypsin: cuts C-term side of KR unless next residue is P
Sequence Coverage: 73%

Matched peptides shown in **Bold Red**

```
  1 MMGLGNGRRS MKSPPLVLAA LVACIIVLGF NYWIASSRSV DLQTRIMELE
 51 GRVRRAAAER GAVELKKNEF QGELEKQREQ LDKIQSSHNF QLESVNKLYQ
101 DEKAVLVNNI TTGERLIRVL QDQLKTLQRN YGRLQQDVLQ FQKNQTNLER
151 KFSYDLSQCI NQMKEVKEQC EERIEEVTKK GNEAVASRDL SENNDQRQQL
201 QALSEPQPRL QAAGLPHTEV PQGKGNVLGN SKSQTPAPSS EVVLDSKRQV
251 EKEETNEIQV VNEEPQRDRL PQEPGREQVV EDRPVGGRGF GGAGELGQTP
301 QVQAALSVSQ ENPEMEGPER DQLVIPDGQE EEQEAAGEGR NQQKLRGEDD
351 YNMDENEAES ETDKQAALAG NDRNIDVFNV EDQKRDTINL LDQREKRNHT
401 L
```

Supplementary Figure S1: Mascot search result confirmed these protein gels correspond to GP73.

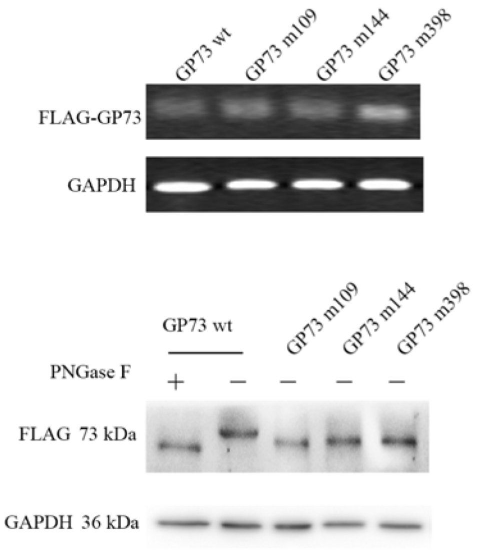

Supplementary Figure S2: Construction and confirmation of GP73 N-glycosylation site deletion mutants: GP73 m109 (Asn109 mutated to Gln109), GP73 m144 (Asn144 mutated to Gln144), and GP73 m398 (Asn398 mutated to Gln398).

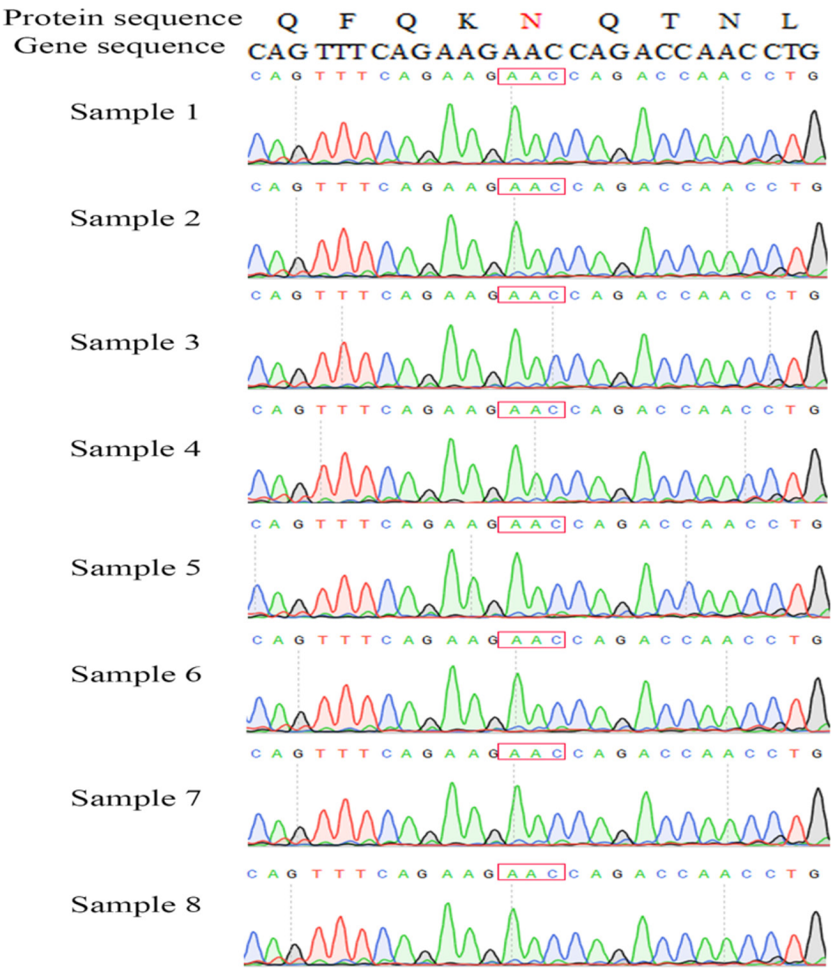

Supplementary Figure S3: Eight samples from HCC tissues were collected and sequenced to determine the mutation state of GP73 at Asn144.
